# The Employment of Dentists in the Army

**Published:** 1859-06

**Authors:** 


### THE EMPLOYMENT OF DENTISTS IN THE ARMY.

THIS proposition, as may be remembered, was brought prominently to the notice of the profession, through the report of the proceedings of the last meeting of the American Dental Convention.

That it may not be lost sight of, we republish the resolutions as passed, with the names of committees appointed, as follows:

"Dr. McKellops offered the following preamble and resolution:

*Whereas*, Owing to the great inconvenience of the officers and soldiers in procuring competent dentists, when necessarily required, and knowing the difficulty in which they are placed, being stationed at distant posts, where it is absolutely impracticable for a regular practitioner of dentistry to visit them; therefore,

"*Resolved*, That this Society appoint a committee of five, for the purpose of memorializing Congress on the necessity of appointing dentists to be attached to the regular army, and that we recommend the same to the consideration of the American Dental Convention, and ask their coöperation with us.

"The resolution was adopted, and the following committee appointed: Drs. McKellops, Spalding, Forbes, Branch, and Lewis. President Allport added by vote.

"By Dr. McKellops—

"*Resolved*, That the Convention appoint a committee of five, to memorialize Congress, on the necessity of appointing dentists for service in the regular army. The committee to act in concert with that of the 'Western Dental Society.'

"Committee—Dr. James Taylor, Cincinnati, O.; Dr. Geo. Watt, Xenia, O.; Dr. Frank Fuller, Portsmouth, N. H.; Dr. W. H. Atkinson, Cleveland, O.; Dr. Stone, Lexington, Ky."

The mover of the resolutions, in both the Dental Conventions, has "seen some service," and has, no doubt, been painfully impressed with the sufferings endured by the poor fellows composing our army in Mexico, and if such were the case with an army, composed largely of volunteers, what must it be with the professional soldier, who has but rarely the opportunity, even had he the ability, to employ the services of the dentist.

As to their condition and necessities, take for example the army in Utah, and particularly during their fatiguing and lengthy march through the wilderness, and their exposed encampments on the route, also the other portions of the army scattered along our frontier, their only neighbors the Indians.

We are almost disposed to say, after careful reflection, that the soldier has as much need of a dentist as for a surgean, and that in the aggregate, he suffers as much or more from diseases of the teeth as from any cause incident to his profession. He certainly has as much use for his teeth as for his limbs, and we are quite sure he does more eating than fighting, and should, therefore, be as well furnished with the needful appliances for the effective performance of the former, as he is for the latter, and as his occupation is a hard one at best, he is deserving and entitled to as many comforts and as much exemption from annoyance and suffering as it is possible to afford him.

For these and other considerations which might be presented, we esteem the matter one of very great importance and can not but admire the humanity in such a proposition, and wonder that it was never made before.

In this connection, we would refer our readers to some extracts (which will be found in the *Dental News Letter*, for July, 1857), made from "an Army Medical Circular," issued by the Medical Director General of the British army, calling the attention of the medical staff to the improvements made in dentistry, in which he says:

"I am of opinion that a considerable gain to the service, besides comfort to individuals, would accrue from a more improved practice in dental surgery than that which has hitherto obtained in military life."

And again, after referring to the nature of these improvements, and their introduction in the army, says, that "if carefully practiced in the service, will exercise, I feel convinced, a not unimportant influence in the efficiency of regiments; will obviate the necessity of having recourse to the forceps in numerous cases, and will add to the comfort and health of the soldier."

We sincerely trust that the committees which have been appointed, will be enabled, by a vigorous prosecution of the matter before the proper authorities, to bring it to a successful termination.—*Dental News Letter.*

